# Psychological Inflexibility in Depression with Psychotic Features: A Case Report

**DOI:** 10.1192/j.eurpsy.2023.2180

**Published:** 2023-07-19

**Authors:** S. Akyildirim Cor, S. Yetkin

**Affiliations:** 1 Psychiatry, Gulhane School of Medicine, Ankara, Türkiye

## Abstract

**Introduction:**

Major depressive disorder (MDD) is a mood disorder that can last for weeks or even months, in which there is a depressed mood accompanied by anxiety, in addition to negative changes in cognitive functions, psychomotor movement and vegetative functions. Depression with psychotic features is a psychiatric syndrome that progresses with delusions as well as severe symptoms such as psychomotor retardation or agitation, depressive ruminations, deterioration in cognitive functions, and confusion. Compared to the subtypes without psychotic features, the symptoms are more severe, the age of onset is earlier, and the duration of the disease is longer. Feelings of guilt, worthlessness and suicidal thoughts and attempts at suicide are more common. The risk of exacerbation is greater. Diagnosis of bipolar disorder and schizophrenia is more common in first-degree relatives of these patients.

**Objectives:**

An 18-year-old female patient with somatic delusions and psychotic persistence that started after a sexual trauma and persisted for 1 month was consulted after organic exclusions were made. It is understood from the anamnesis that the patient had a manic episode about 6 months ago and that his mother was followed up with a diagnosis of bipolar disorder. The patient’s current clinical picture was evaluated as depression with psychotic features, and after hospitalization, the treatment was adjusted as fluoxetine 20 mg/g, olanzapine 5 mg/g, and lithium 900 mg/g. Self as context, cognitive defusion and acceptance interventions were applied to the patient.

**Methods:**

When the Cognitive Fusion Questionnaire(CFQ), Self-as-Context Scale(SACS), Acceptance and Action Questionnaire(AAQ-II) completed by the patient during hospitalization and in remission periods were compared, it was observed that there was a significant regression in the patient’s psychological inflexibility during the period of remission. Written informed consent was obtained from the patient whose clinical picture was presented in order to contribute to the scientific literature.

**Results:**

Depression with psychotic features is another clinical picture in which psychological inflexibility increases, and it has been observed that interviews to increase psychological flexibility during the treatment process contribute positively to the treatment process. For this reason, the contribution to the healing process can be better clarified in further studies on interventions to increase psychological flexibility applied in addition to pharmacological treatments.

**Image:**

**Figure:**
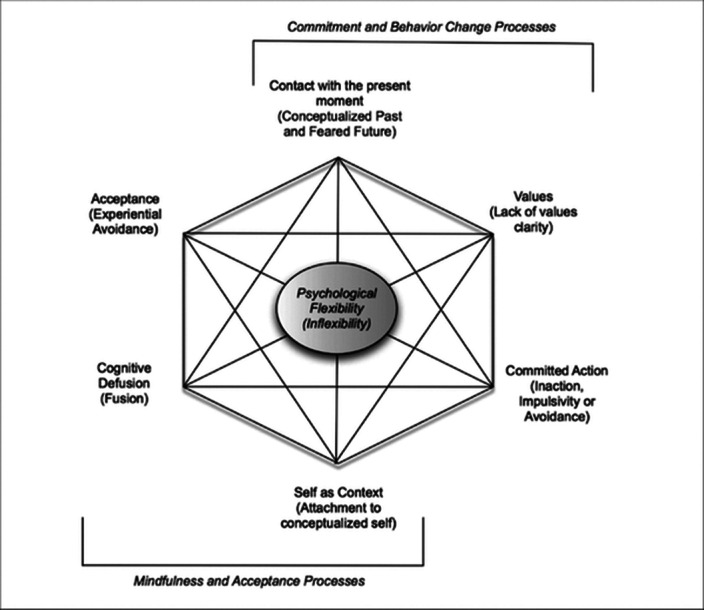

**Conclusions:**

Psychological inflexibility is an effort to control a person’s emotion, thought, behavior or experience in a dysfunctional way in the face of an undesired experience. It has been seen in studies conducted in recent years that; There is a significant positive correlation between high psychological inflexibility and somatization, depression, anxiety and other psychological disorders.

**Disclosure of Interest:**

None Declared

